# Kelulut Honey Modulates Skeletal Health and Bone-Related Molecular Markers in Rats Fed a High-Carbohydrate and High-Fat Diet

**DOI:** 10.3390/life16091419

**Published:** 2026-08-26

**Authors:** Sophia Ogechi Ekeuku, Kumeshini Sukalingam, Mohd Fahami Nur Azlina, Fairus Ahmad, Kok-Yong Chin, Elvy Suhana Mohd Ramli

**Affiliations:** 1 Department of Pharmacology, Faculty of Medicine, Universiti Kebangsaan Malaysia, Cheras, Kuala Lumpur 56000, Malaysia; sogechie@ukm.edu.my (S.O.E.); nurazlinamf@ukm.edu.my (M.F.N.A.); chinky@ukm.edu.my (K.-Y.C.); 2 Department of Anatomy, Faculty of Medicine, Universiti Kebangsaan Malaysia, Cheras, Kuala Lumpur 56000, Malaysia; meshni_anat@yahoo.com.my (K.S.); fairusahmad@ukm.edu.my (F.A.)

**Keywords:** Kelulut honey, high-carbohydrate high-fat diet, bone remodeling, oxidative stress, bone mineral density

## Abstract

Background: High-carbohydrate high-fat (HCHF) diets have been associated with oxidative stress, impaired bone remodelling, and deterioration of skeletal integrity. Kelulut honey (KH), a stingless bee honey rich in antioxidant compounds, has demonstrated protective effects against bone loss; however, its effects on bone density, biomechanical properties, antioxidant status, and bone-related molecular markers remain incompletely understood. (2) Methods: Male Wistar rats were assigned to sham, negative control (NC), or KH-treated groups (200, 400, and 600 mg/kg). The NC and KH groups received an HCHF diet for 24 weeks, with KH administered during the final 12 weeks. Bone microarchitecture was evaluated using micro-computed tomography, bone mineral density (BMD) by dual-energy X-ray absorptiometry, and biomechanical properties by three-point bending. Antioxidant enzyme activities, serum bone turnover markers, and bone-related gene expression were also assessed. (3) Results: HCHF feeding reduced BMD, load, stress, Young’s modulus, gluthatione peroxidase (GPx) activity, and superoxide dismutase (SOD) activity while increasing strain, displacement, serum C-terminal telopeptide of type 1 collagen (CTX-1) and receptor activator of nuclear factor-κB (RANK) levels, and runt-related transcription factor 2 (*Runx2*) expression. KH supplementation improved bone health by dose-dependently enhancing antioxidant defences, significantly increasing GPx and SOD activities, while catalase (CAT) activity was elevated at 600 mg/kg. The 400 mg/kg dose improved trabecular microarchitecture by increasing trabecular number, connectivity density and cortical area/total tissue area. KH also improved biomechanical properties, increasing load, stress, stiffness, and Young’s modulus while reducing displacement. Furthermore, KH attenuated HCHF-induced increases in *Runx2* mRNA and RUNX2 protein and reduced osterix (*Sp7*), integrin Subunit Alpha 5 (*Itga5*), and calcitonin receptor (*Calcr)* mRNA expression. However, serum CTX-1 remained elevated in all KH-treated groups. (4) Conclusions: Overall, the findings suggest that KH may have potential as a natural intervention for mitigating diet-associated skeletal alterations, although further studies are required to clarify the underlying mechanisms.

## 1. Introduction

The maintenance of bone health is a complex physiological process that relies on the delicate balance between bone formation by osteoblasts and bone resorption by osteoclasts [1]. Modern dietary patterns, specifically those high in carbohydrates and fats (HCHF), have been increasingly recognized as significant contributors to bone loss and the deterioration of skeletal integrity [2]. Previous studies on animal models showed that prolonged consumption of an HCHF diet leads to significant reductions in bone mineral density (BMD) and bone mineral content (BMC) across the whole body, including the femur [3,4]. This diet-induced skeletal decline is primarily driven by chronic inflammation and oxidative stress, which trigger an imbalance in bone remodeling by promoting osteoclast activity while suppressing osteogenic differentiation [5,6].

One of the critical mechanisms underlying HCHF-induced bone loss is the expansion of bone marrow adiposity. Since osteoblasts and adipocytes originate from the same pool of mesenchymal stem cells, an HCHF environment often favors adipocyte differentiation at the expense of bone-forming cells, thereby depleting the capacity for bone formation and contributing to a weakened skeletal structure [7]. Furthermore, the resulting oxidative stress, characterized by increased reactive oxygen species and weakened antioxidant defenses, creates a pro-resorptive environment that further compromises bone strength [8].

Kelulut honey (KH), a unique functional food produced by stingless bees (Heterotrigona itama), has emerged as a potential natural intervention for preserving bone health due to its high polyphenol and phenolic acid content. A previous study established that KH possessed potent antioxidant properties, which protected bone microarchitecture in glucocorticoid-induced bone loss. In our earlier investigations, we demonstrated that KH supplementation effectively prevented the increase in osteoclast surfaces in rats subjected to an HCHF diet [2]. Furthermore, we recently reported that KH can suppress HCHF-induced marrow adiposity and improve bone flexibility by increasing displacement and strain resistance [9].

Despite these findings, comprehensive data linking the skeletal protective effects of KH to specific densitometric changes, detailed biomechanical strength profiles, and the underlying molecular mechanisms remain incomplete. While our previous work highlighted microarchitectural and cellular improvements, the impact of KH on systemic redox status and the specific expression of bone-forming genes in an HCHF-induced model remains to be elucidated. Therefore, this study aims to expand upon our published findings by evaluating the effects of KH on femoral BMD, intrinsic and extrinsic biomechanical parameters, and the skeletal antioxidant environment. Most importantly, this manuscript investigates how KH modulates the expression of critical bone markers, such as type I collagen (*Col1a1*) and osteoprotegerin (*Tnfrsf11b*), providing a definitive molecular basis for its role in mitigating diet-induced bone loss.

Previous studies from our group have characterized several skeletal consequences of HCHF feeding over a 16-week experimental period, including alterations in bone microarchitecture, biomechanical properties, histological features, antioxidant status, and selected bone-related gene expression. However, the present study was conducted using a completely independent animal cohort and a longer 24-week experimental period. None of the animals, biological samples, or datasets reported in the present study were included in our previous publications. Although selected endpoints, including micro-CT, three-point bending, and antioxidant enzyme activities, were similarly assessed, these measurements represent newly generated data from the independent cohort. The present study further extends the previous work by incorporating serum biochemical assessment, and a broader panel of osteogenic and bone-remodelling markers, including runt-related transcription factor 2 (*Runx2*), osterix (*Sp7*), integrin Subunit Alpha 5 (*Itga5*), bone gamma-carboxyglutamate protein (*Bglap*), cathepsin K (*Ctsk*), Calcitonin receptor (*Caclr*), tumor necrosis factor ligand superfamily member 11 (Tnfrsf11), *Col1a1* and *Tnfrsf11b*. The longer experimental duration also provides an opportunity to evaluate the effects of HCHF feeding and KH supplementation over 24 weeks. Collectively, this study provides an independent and extended assessment of the effects of KH on skeletal health in HCHF-fed rats.

## 2. Materials and Methods

### 2.1. Preparation of KH for Animal Treatment

Raw honey (KH) sourced from the stingless bee species *Heterotrigona itama* was acquired from a local apiary located in Gombak, Selangor, Malaysia. It was kept in a glass container at 4 °C. The same KH material used in the present study was previously characterized for its bioactive compounds by Ekeuku et al. [9]; therefore, the detailed phytochemical composition is not repeated here (Table S1). No formal batch or lot number was assigned to the KH material. Prior to oral gavage administration, KH was mixed with distilled water at a 1:1 ratio and administered according to the designated experimental dose.

### 2.2. HCHF Preparation

The HCHF diet was prepared per kilogram using 395 g of sweetened condensed milk (Fraser & Neave Holdings Bhd., Kuala Lumpur, Malaysia), 200 g of ghee (Enrico’s Pure Ghee, Raviraj Sdn. Bhd., Penang, Malaysia), 175 g of D-(−)-fructose (Emprove^®^ Essential, Merck, Darmstadt, IN, USA), 155 g of powdered rat chow (Gold Coin Feedmills (M) Sdn. Bhd., Selangor, Malaysia), 25 g of Hubble, Mendel, and Wakeman salt mixture (MP Biomedicals, Santa Ana, CA, USA), and 50 mL of water. Rats assigned to the HCHF groups additionally received drinking water containing 25% fructose (Merck). This dietary regimen has previously been demonstrated to induce metabolic syndrome (MetS) in rats following 16 weeks of exposure [10]. Feed and drinking water were provided ad libitum throughout the experimental period.

### 2.3. Animals

Thirty-five 12-week-old male Wistar rats were obtained from the Laboratory Animal Resource Unit, Universiti Kebangsaan Malaysia (Kuala Lumpur, Malaysia). Upon arrival, the animals were housed individually under standard laboratory conditions at 23 ± 2 °C and allowed to acclimatize for 1 week. A 12-h light/12-h dark cycle was maintained throughout the acclimatization and experimental periods. All experimental procedures were conducted in accordance with the animal research guidelines of Universiti Kebangsaan Malaysia and were approved by the Universiti Kebangsaan Malaysia Animal Ethics Committee (ANAT/FP/2020/ELVYSUHANA/25-MAR./1090-MAR.-2020-JAN.-2023).

### 2.4. Randomisation and Blinding

Following acclimatization, animals were randomly allocated to the experimental groups using a random-number method. Allocation was performed before baseline body-weight measurements and without weight-based stratification. Personnel conducting the animal procedures were aware of treatment allocation. Data analysts were not involved in animal handling or experimental measurements and received the raw data for statistical analysis.

### 2.5. Study Design

Following acclimatization, the rats were randomly allocated to five experimental groups (*n* = 7 per group): sham, negative control (NC), and three Kelulut honey (KH) treatment groups receiving 200, 400, or 600 mg/kg (200 KH, 400 KH, and 600 KH, respectively). The sham group received standard rodent pellets (Gold Coin, Klang, Malaysia) and tap water ad libitum, whereas the NC and KH groups were maintained on the HCHF diet with 25% fructose-supplemented drinking water for 24 weeks. During the final 12 weeks, rats in the KH groups received KH orally by gavage at the respective doses, while the sham and NC groups received distilled water. The establishment of metabolic syndrome (MetS) in this experimental model has been described previously [10]. At the end of the 24-week intervention, the animals were euthanized using an overdose of a ketamine/xylazine/Zoletil mixture (0.3 mL/100 g body weight). Blood was collected for serum preparation, after which both femora and tibiae were excised and carefully cleared of adherent soft tissue. The left and right femora and tibiae were subsequently stored at −80 °C for biochemical analyses.

### 2.6. Micro-Architectural Indices of Rats

Micro-computed tomography (micro-CT) was performed using a SkyScan 1076 system (Scanco Medical, Brüttisellen, Switzerland) to assess cortical and trabecular bone microarchitecture. The left tibia was positioned securely in the specimen holder and scanned at 85 kV and 170 µA, with a 0.5-mm aluminium filter and a rotation step of 0.3°. Images were acquired at an isotropic pixel size of 17.85 µm using medium camera resolution, with frame averaging set to 3 and an integration time of 400 ms. Image reconstruction was performed using NRecon software (v1.6.10.4, SkyScan, Kontich, Belgium). For trabecular bone analysis, the volume of interest (VOI) was defined beginning 1.0 mm distal to the proximal growth plate, while the cortical VOI began 5.0 mm distal to the proximal growth plate and extended to the distal end of the tibia. Three-dimensional image reconstruction and quantitative analysis of bone microarchitecture were conducted using CTAn software (v1.16.1.0+, SkyScan, Belgium). Trabecular parameters included bone volume fraction (BV/TV), trabecular separation (Tb.Sp), trabecular thickness (Tb.Th), trabecular number (Tb.N), connectivity density (Conn.D), and structure model index (SMI). Cortical parameters assessed were cortical area fraction (Ct.Ar/Tt.Ar), cortical thickness (Ct.Th), cortical area (Ct.Ar), and total tissue area (Tt.Ar).

### 2.7. Measurement of Bone Mineral Density

Bone mineral density (BMD) was assessed by dual-energy X-ray absorptiometry (DXA) under general anaesthesia. Anaesthetised rats were positioned in ventral recumbency on the scanning table, and DXA scans were performed at weeks 0, 12, and 24. BMD measurements were analysed using the manufacturer-recommended Small Animal Analysis Software on the Hologic QDR-1000 system (Version 5.52).

### 2.8. Measurement of Bone Biomechanical Strength Parameters

The biomechanical properties of the femurs were assessed using an Instron Universal Testing Machine (model 5560; Instron, Canton, MA, USA) equipped with Bluehill 2 software (Instron, Canton, MA, USA) in a three-point bending configuration. After thawing to room temperature, the femur length and mid-diaphyseal diameter were measured. Throughout testing, the specimens were kept moist with gauze soaked in phosphate-buffered saline. Each femur was positioned with the anterior surface facing upward and supported at two lower supports with a 10-mm span. A compressive load was applied to the anterior surface at the mid-diaphysis at a displacement rate of 5 mm/min until fracture. Load, displacement, stress, and strain were calculated using Bluehill 2 software based on the specimen dimensions and test configuration parameters. Load–displacement and stress–strain curves were generated from the recorded data. Stiffness was calculated from the slope of the linear portion of the load–displacement curve, while Young’s modulus was determined from the slope of the linear portion of the stress–strain curve. Biomechanical outcomes were categorized as extrinsic properties (load, displacement, and stiffness), reflecting the mechanical behaviour of the whole bone, and intrinsic properties (stress, strain, and Young’s modulus), reflecting the material properties of the bone.

### 2.9. Measurement of Bone Remodelling Parameters

Bone samples were carefully cleaned of adherent soft tissues, snap-frozen in liquid nitrogen, and pulverized into a fine powder using a pre-chilled mortar and pestle. The bone powder was homogenized in RIPA lysis buffer supplemented with a protease inhibitor cocktail and incubated on ice for 30 min. The homogenates were centrifuged at 12,000× *g* for 20 min at 4 °C, and the supernatants were collected for analysis. The concentrations of receptor activator of nuclear factor-κB (RANK) and runt-related transcription factor 2 (RUNX2) were measured using commercially available rat ELISA kits (ELK Biotechnology, Wuhan, China) according to the manufacturer’s instructions, and the results were normalized to the total protein concentration. ELISA technique was used for measuring crosslinked C-telopeptide of type 1 collagen (CTX-1) (Uscn Lifescience Inc., Wuhan, China) directly in the serum per manufacturer’s protocols.

### 2.10. Lipid Peroxidation and Oxidative Stress Enzymes

The lateral halves of the left femurs were used to measure oxidative stress enzyme activities and lipid peroxidation. The homogenates were prepared according to the manufacturers’ procedures and guidelines. The superoxide dismutase (SOD), catalase (CAT) and glutathione peroxidase (GPx) were quantified using the microtiter kits (Cayman Chemical Company, Ann Arbor, MI, USA).

### 2.11. Bone Formation and Bone Resorption-Related Gene Expressions

Bone homogenates were obtained from trabecular bone samples taken from the distal part of the right femur. Each sample was placed into a tube containing steel beads, which were homogenised in the manufacturer-provided buffer using a high-speed homogeniser (Bead Ruptor 24, Omni, Kennesaw, GA, USA) at 4 °C. The tissue lysate was incubated at 65 °C and centrifuged to precipitate the debris. The QuantiGene Plex 2.0^®^ technique (Panomics/Affymetrix Inc, Santa Clara, CA, USA) was used to quantify the mRNA expression. All procedures were performed according to the manufacturer’s instructions. Tissue lysates were pipetted into a 96-well plate preloaded with capture reagent and a probe set and incubated overnight at 54 °C. After incubation, the plate was hybridized with the preamplifier, the amplifier, and the biotinylated label. Oligonucleotide probe sets used were designed by the manufacturer. A Luminex^®^ instrument (Bio-Rad, Hercules, CA, USA) was used to measure luminescence, and the mean fluorescence intensity specific to each gene (proportional to the mRNA captured by the bead) was calculated. Expression of each gene was normalized to glyceraldehyde-3-phosphate dehydrogenase. Table 1 shows the analysed genes and there NCBI gene IDs.

### 2.12. Statistical Analysis

Statistical analysis was conducted using version 26 of the Statistical Package for Social Sciences (SPSS) (IBM, Armonk, NY, USA). All animal data points were included in the analysis. The Shapiro-Wilk test was employed to assess data normality, confirming that all datasets followed a normal distribution. Mixed design analysis of variance (ANOVA) with small effect analysis as post hoc test was used to evaluate mean differences in body weight and BMD data while one-way ANOVA with Tukey’s pairwise comparison was utilized to evaluate mean differences among other study groups, with statistical significance set at *p* < 0.05.

## 3. Results

### 3.1. Body Weight Changes

**Figure 1:**
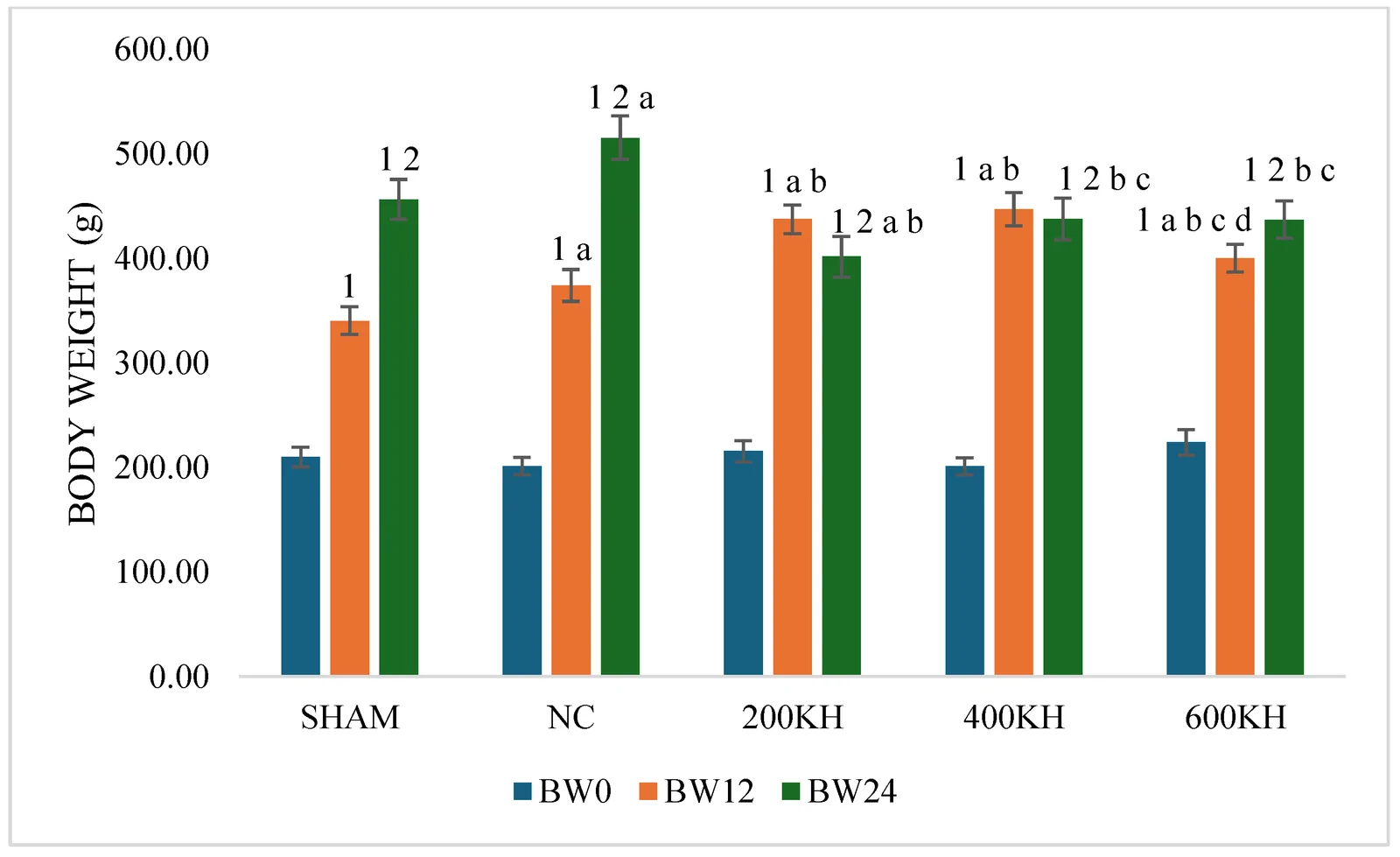
Body weight changes. Data are expressed as mean ± SD (*n* = 7 per group). Statistical significance was evaluated by mixed design analysis of variance (ANOVA) with small effect analysis as post hoc test. ^a^ *p* < 0.05 vs. SHAM, ^b^ *p* < 0.05 vs. NC, ^c^ *p* < 0.05 vs. 200 KH, ^d^ *p* < 0.05 vs. 400 KH at same time-point; ^1^ *p* < 0.05 vs. BW0, ^2^ *p* < 0.05 vs. BW12 of same group.

Body weight increased significantly from baseline (week 0) to week 12 in all groups (*p* < 0.05). In the sham, negative control (NC), and 600 KH groups, body weight continued to increase significantly at week 24 compared with week 12 (*p* < 0.05). In contrast, the 200 KH and 400 KH groups exhibited a significant decrease in body weight at week 24 relative to week 12 (*p* < 0.05), although body weight remained significantly higher than baseline. Between-group comparisons showed that the NC group had significantly higher body weight than the sham group at both weeks 12 and 24 (*p* < 0.05). At week 12, all KH-treated groups exhibited significantly higher body weight than the sham and NC groups (*p* < 0.05). At week 24, the 200 KH group exhibited significantly lower body weight than the sham and NC groups (*p* < 0.05), whereas the 400 KH and 600 KH groups showed significantly lower body weight than the NC group (*p* < 0.05). At week 12, body weight in the 600 KH group was significantly lower than that in the 200 KH and 400 KH groups (*p* < 0.05). By week 24, body weight in the 400 KH and 600 KH groups was significantly higher than that in the 200 KH group (*p* < 0.05) (Figure 1).

### 3.2. Micro-Architectural Indices of Rats

**Figure 2:**
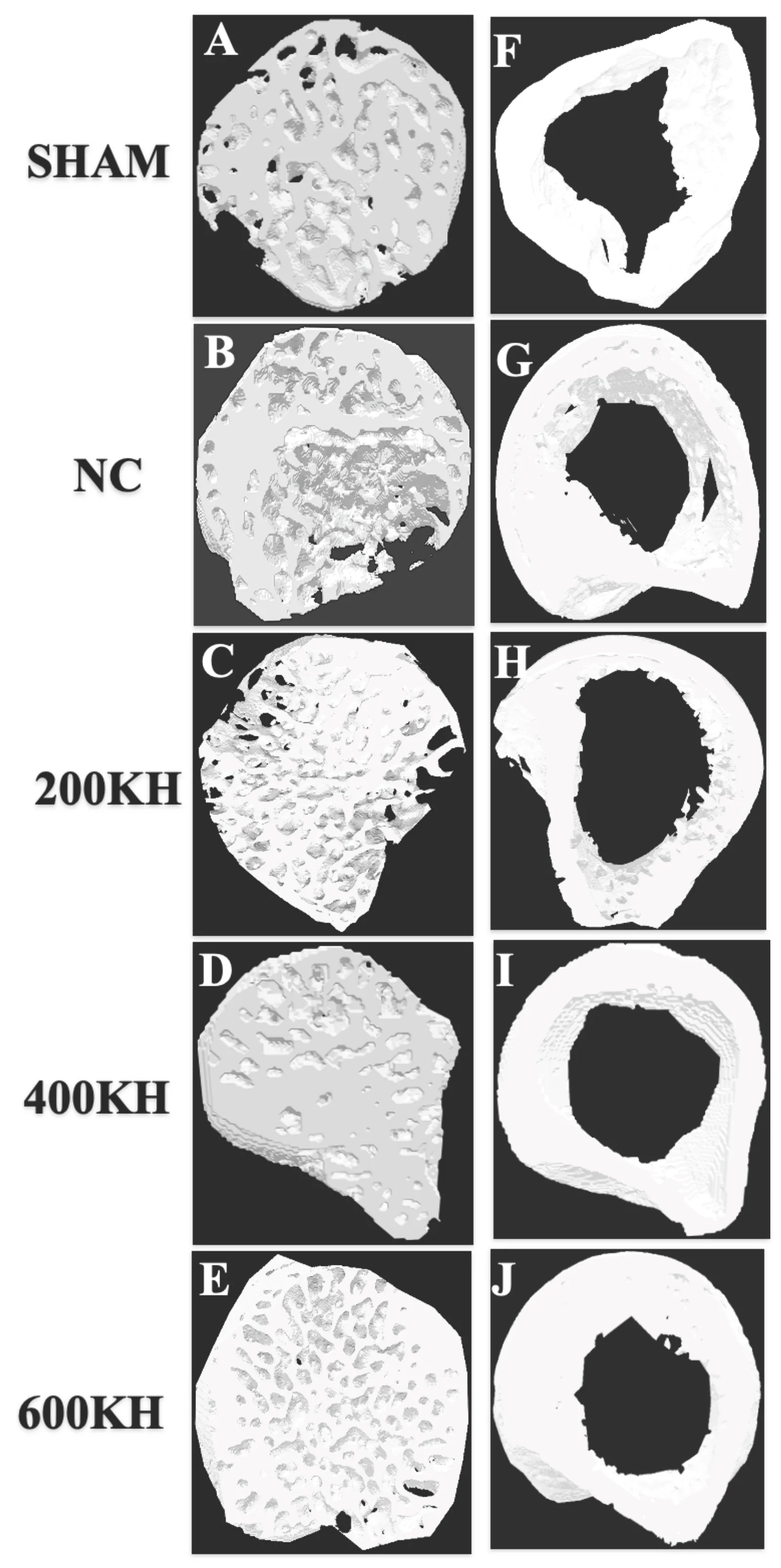
3D images of trabecular (**A**–**E**) and cortical (**F**–**J**) part of left tibia obtained from microCT scanning.

**Figure 3:**
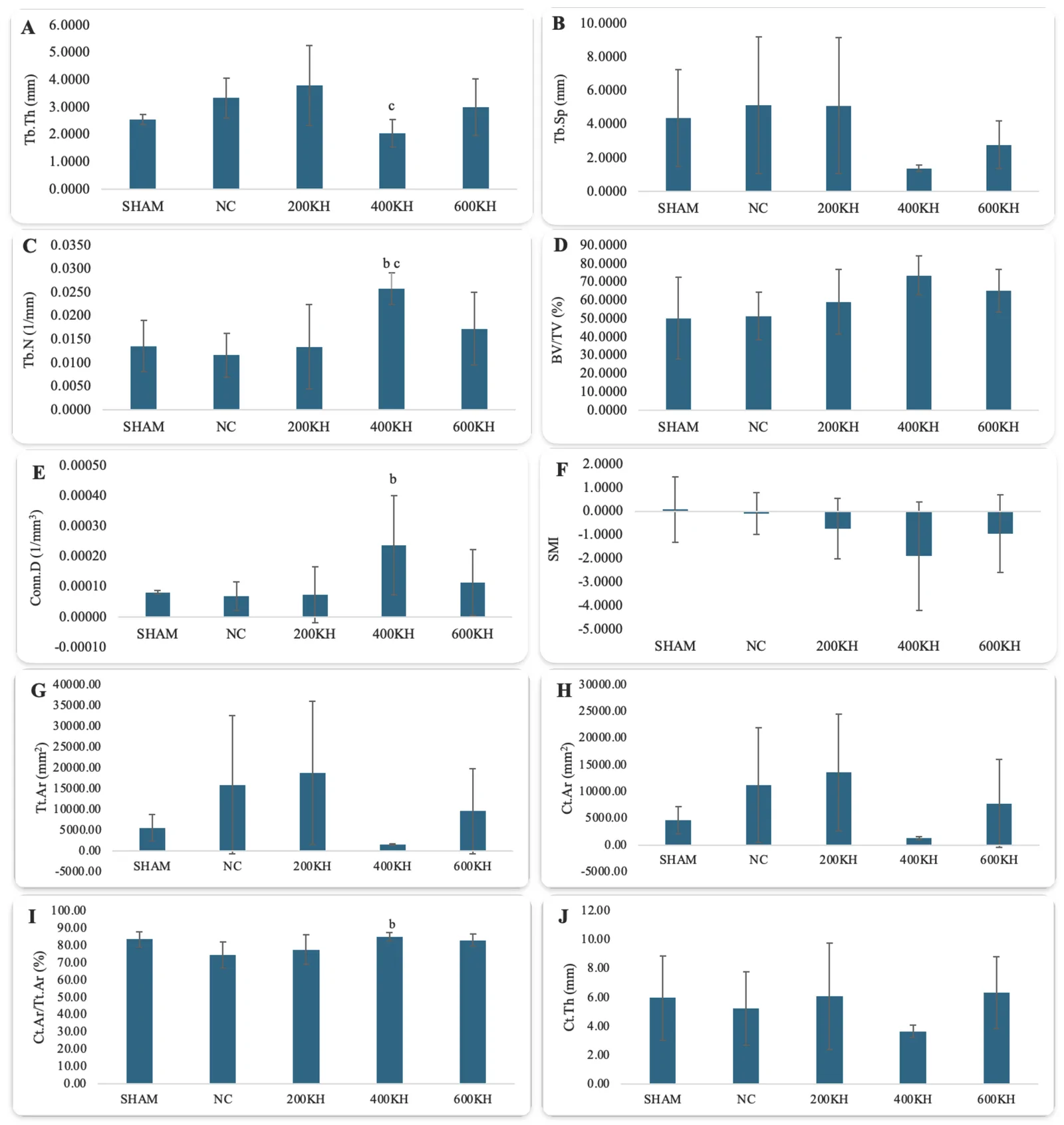
Structural indices of femur bone evaluated (**A**–**J**). Data are expressed as mean ± SD (*n* = 7 per group). Statistical significance was evaluated by one-way ANOVA followed by Tukey’s HSD multiple-comparison test. ^b^ *p* < 0.05 vs. NC, ^c^ *p* < 0.05 vs. 200 KH.

Microarchitectural analysis revealed that 24 weeks exposure to HCHF had no significant effect on microarchitecture parameters (*p* > 0.05) (Figure 2A–J). However, the 400 KH group exhibited a significantly higher Tb.N compared with both the sham and NC groups (*p* < 0.05) (Figure 3C). Similarly, Conn.D was significantly increased in the 400 KH group relative to the NC group (*p* < 0.05) (Figure 3E). In contrast, Tb.Th was significantly reduced in the 400 KH group compared with the 200 KH group (*p* < 0.05) (Figure 3A). Furthermore, Ct.Ar/Tt.Ar was significantly higher in the 400 KH group than in the NC (*p* < 0.05) (Figure 3I). No significant differences were observed after KH supplementation among the experimental groups for Tb.Sp (Figure 3B), BV/TV (Figure 3D), SMI (Figure 3F), Tt.Ar (Figure 3G), Ct.Ar (Figure 3H) and Ct.Th (Figure 3J) (*p* > 0.05).

### 3.3. Bone Densitometric and Biomechanical Assessment

**Figure 4:**
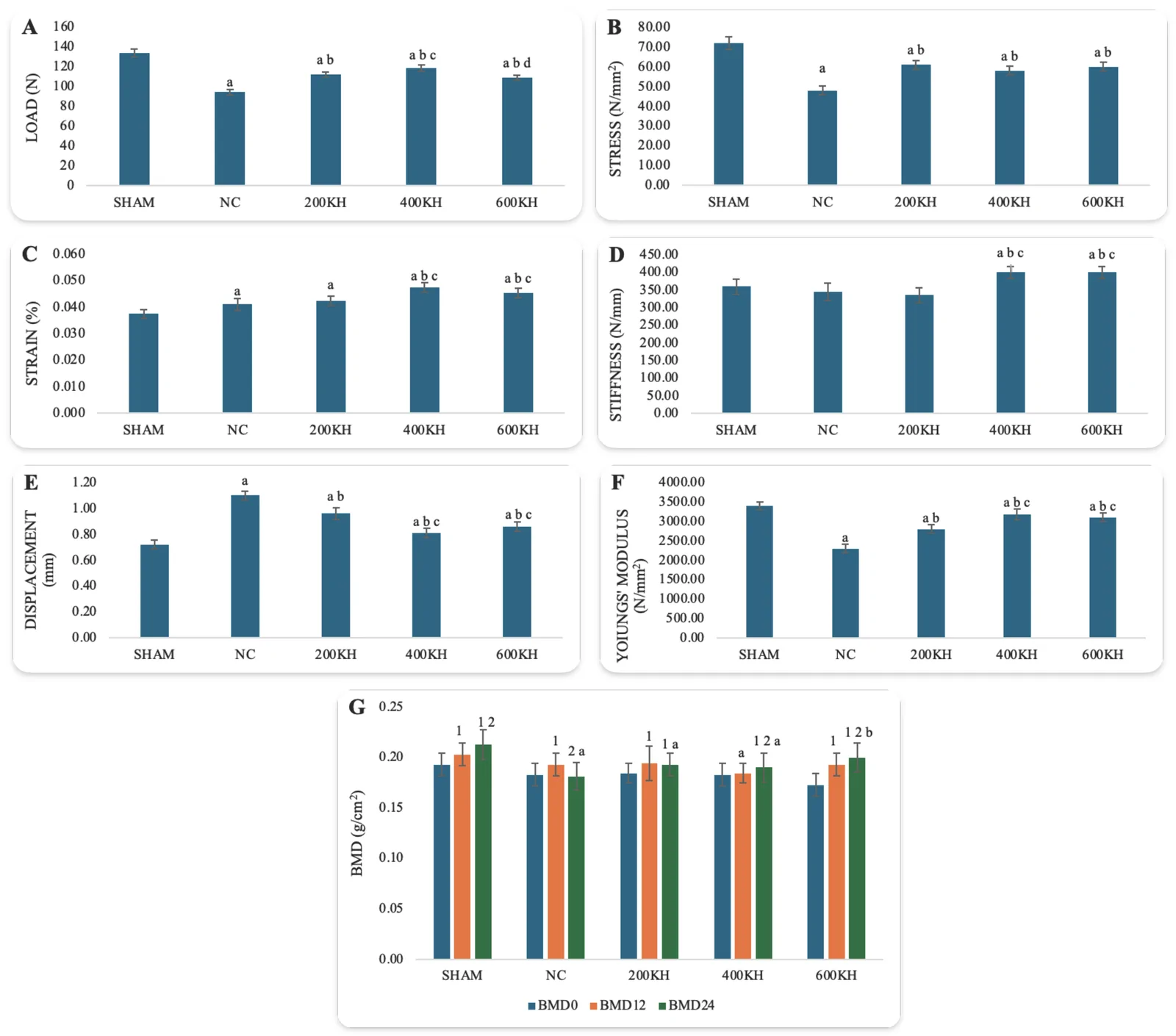
Biomechanical indices. Data are expressed as mean ± SD (*n* = 7 per group). Statistical significance was evaluated by one-way ANOVA followed by Tukey’s HSD multiple-comparison test. ^a^ *p* < 0.05 vs. SHAM, ^b^ *p* < 0.05 vs. NC, ^c^ *p* < 0.05 vs. 200 KH at same time-point, ^d^ *p* < 0.05 vs. 400 KH; ^1^ *p* < 0.05 vs. BMD0, ^2^ *p* < 0.05 vs. BMD12 of same group.

Biomechanical analysis revealed significant alterations in bone strength parameters across the experimental groups. Load values were significantly lower in the NC, 200 KH, 400 KH, and 600 KH groups compared with the sham group (*p* < 0.05). However, all KH-treated groups exhibited significantly higher load values than the NC group (*p* < 0.05), with the 400 KH group showing a further increase compared with the 200 KH group, while the 600 KH group demonstrated a lower load value than the 400 KH group (*p* < 0.05) (Figure 4A). Similarly, stress values were significantly reduced in the NC and all KH-treated groups relative to the sham group (*p* < 0.05), although treatment with 200 KH, 400 KH, and 600 KH significantly improved stress compared with the NC group (*p* < 0.05) (Figure 4B). Strain was significantly increased in the NC and all KH-treated groups compared with the sham group (*p* < 0.05), whereas the 400 KH and 600 KH groups exhibited significantly greater strain than both the NC and 200 KH groups (*p* < 0.05) (Figure 4C). Stiffness was significantly higher in the 400 KH and 600 KH groups compared with the sham, NC, and 200 KH groups (*p* < 0.05) (Figure 4D). Displacement was significantly elevated in the NC and all KH-treated groups relative to the sham group (*p* < 0.05); however, all KH-treated groups showed significantly lower displacement than the NC group (*p* < 0.05), with the 400 KH and 600 KH groups also demonstrating lower values than the 200 KH group (*p* < 0.05) (Figure 4E). In contrast, Youngs’ modulus was significantly reduced in the NC and all KH-treated groups compared with the sham group (*p* < 0.05), although treatment with 200 KH, 400 KH, and 600 KH significantly increased Youngs’ modulus relative to the NC group (*p* < 0.05), with the 400 KH and 600 KH groups exhibiting further improvements compared with the 200 KH group (*p* < 0.05) (Figure 4F).

BMD increased significantly in the sham group at weeks 12 and 24 compared with baseline (week 0), with a further increase observed at week 24 relative to week 12 (*p* < 0.05). In the negative control (NC) group, BMD increased at week 12 compared with baseline but declined significantly by week 24 relative to week 12 (*p* < 0.05). The 200 KH group exhibited significantly higher BMD at both weeks 12 and 24 compared with baseline (*p* < 0.05), whereas the 400 KH group showed a significant increase only at week 24 compared with both baseline and week 12 (*p* < 0.05). In the 600 KH group, BMD was significantly elevated at weeks 12 and 24 relative to baseline, with a further increase at week 24 compared with week 12 (*p* < 0.05). Between-group comparisons revealed that the 400 KH group had significantly lower BMD than the sham group at week 12 (*p* < 0.05). At week 24, the NC, 200 KH, and 400 KH groups exhibited significantly lower BMD than the sham group (*p* < 0.05), while the 600 KH group demonstrated significantly higher BMD than the NC group (*p* < 0.05) (Figure 4G).

### 3.4. Skeletal Markers of Redox Status

**Figure 5:**
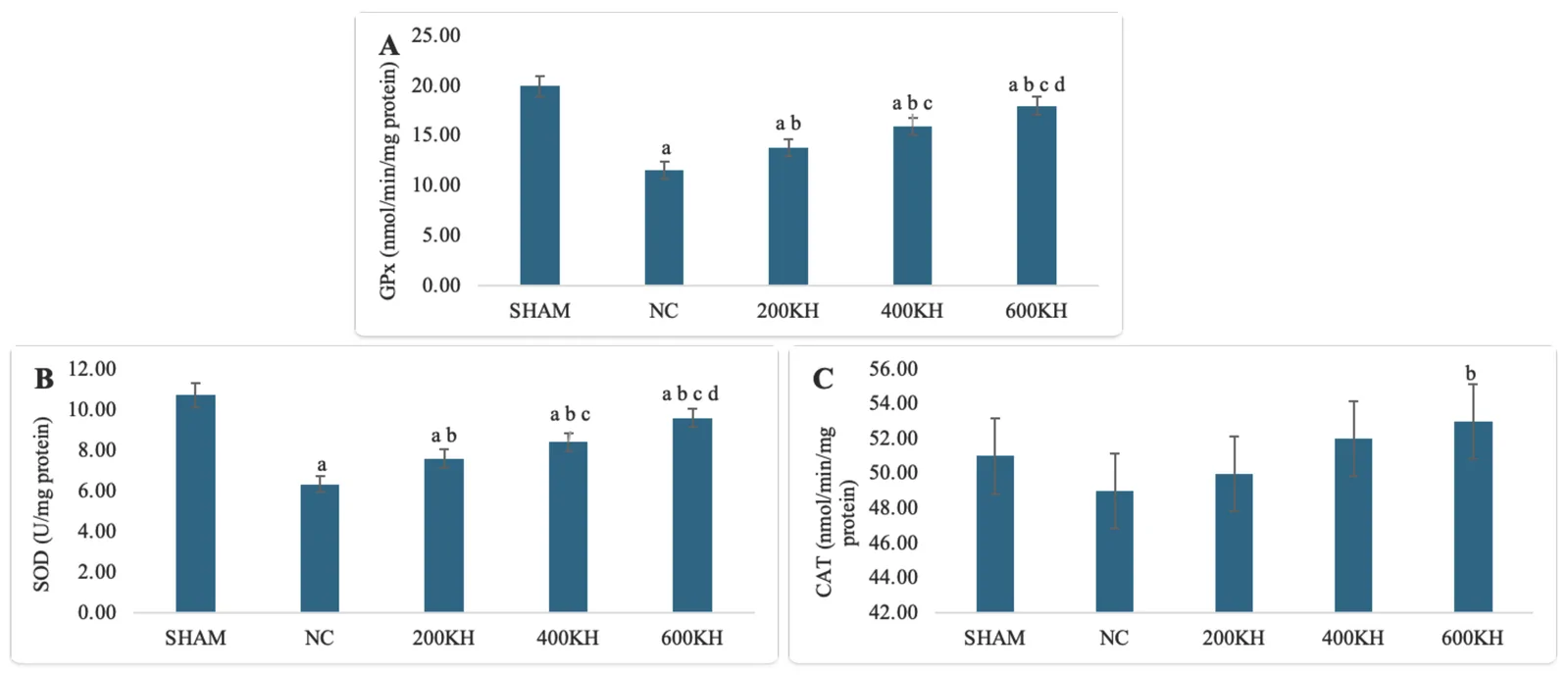
Skeletal markers of redox status*.* Data are expressed as mean ± SD (*n* = 7 per group). Statistical significance was evaluated by one-way ANOVA followed by Tukey’s HSD multiple-comparison test. ^a^ *p* < 0.05 vs. SHAM, ^b^ *p* < 0.05 vs. NC, ^c^ *p* < 0.05 vs. 200 KH, ^d^ *p* < 0.05 vs. 400 KH.

GPx activity was significantly reduced in the NC, 200 KH, 400 KH, and 600 KH groups compared with the sham group (*p* < 0.05). However, all KH-treated groups exhibited significantly higher GPx activity than the NC group (*p* < 0.05). A dose-dependent increase was observed, with the 400 KH group showing significantly higher GPx activity than the 200 KH group, and the 600 KH group exhibiting significantly higher activity than the 400 KH group (*p* < 0.05) (Figure 5A). SOD activity followed a similar pattern. SOD activity was significantly lower in the NC, 200 KH, 400 KH, and 600 KH groups relative to the sham group (*p* < 0.05). Nevertheless, treatment with KH significantly increased SOD activity in the 200 KH, 400 KH, and 600 KH groups compared with the NC group (*p* < 0.05). Furthermore, SOD activity was significantly higher in the 400 KH group than in the 200 KH group and was further increased in the 600 KH group compared with the 400 KH group (*p* < 0.05) (Figure 5B). CAT activity was significantly increased only in the 600 KH group compared with the NC group (*p* < 0.05). No significant differences were observed between the other experimental groups (*p* > 0.05) (Figure 5C).

### 3.5. Bone Remodelling Protein Expression

**Figure 6:**
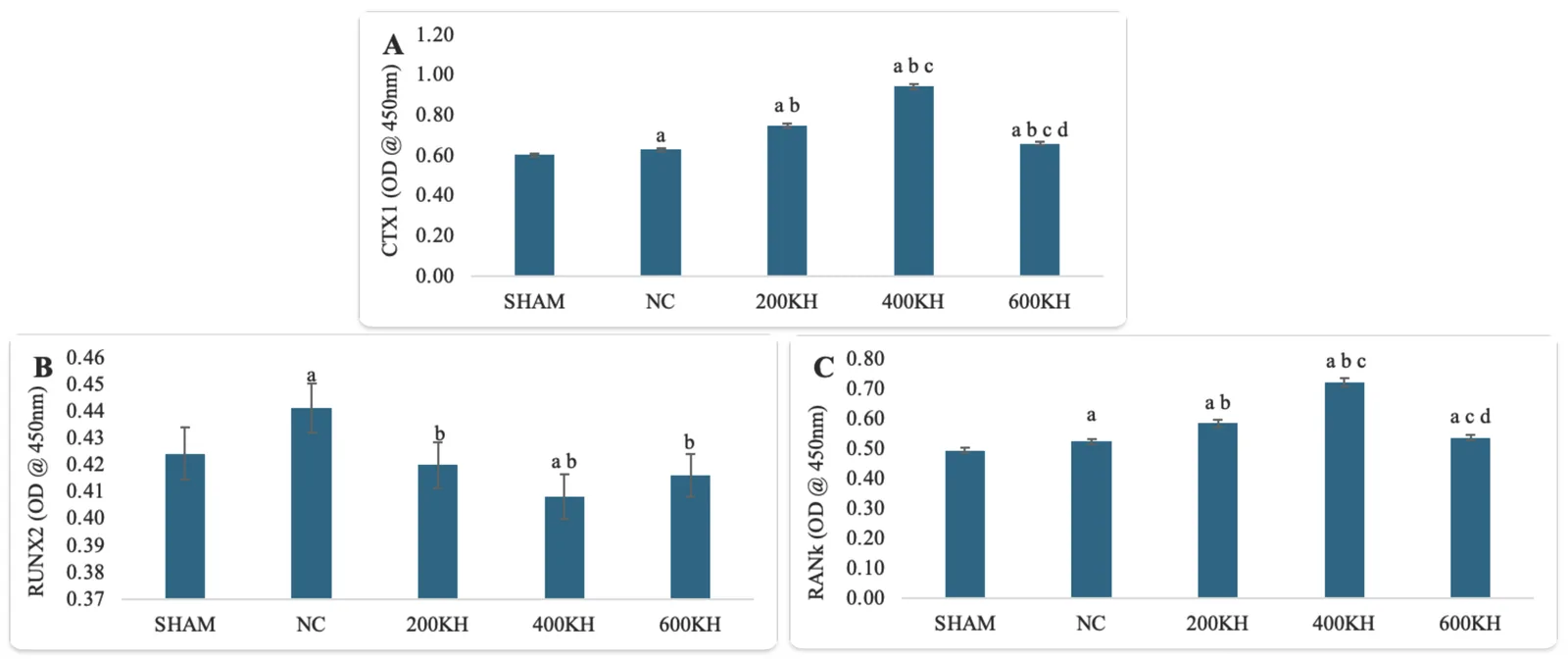
Bone remodelling proteins. Data are expressed as mean ± SD (*n* = 7 per group). Statistical significance was evaluated by one-way ANOVA followed by Tukey’s HSD multiple-comparison test. ^a^ *p* < 0.05 vs. SHAM, ^b^ *p* < 0.05 vs. NC, ^c^ *p* < 0.05 vs. 200 KH, ^d^ *p* < 0.05 vs. 400 KH.

CTX-1 protein levels were significantly elevated in the NC, 200 KH, 400 KH, and 600 KH groups compared with the sham group (*p* < 0.05). Furthermore, all KH-treated groups exhibited significantly higher CTX-1 protein levels than the NC group (*p* < 0.05). Among the treatment groups, the 400 KH group showed significantly higher CTX-1 protein levels than the 200 KH group, whereas the 600 KH group demonstrated significantly lower CTX-1 protein levels than both the 200 KH and 400 KH groups (*p* < 0.05) (Figure 6A). RANK protein levels were significantly increased in the NC, 200 KH, 400 KH, and 600 KH groups relative to the sham group (*p* < 0.05). Compared with the NC group, RANK levels were significantly higher in the 200 KH and 400 KH groups (*p* < 0.05). Additionally, the 400 KH group exhibited significantly greater RANK level than the 200 KH group, while the 600 KH group showed significantly lower RANk level than both the 200 KH and 400 KH groups (*p* < 0.05) (Figure 6B). RUNX2 protein levels were significantly elevated in the NC group compared with the sham group (*p* < 0.05). In contrast, the 400 KH group exhibited significantly lower RUNX2 protein levels than the sham group (*p* < 0.05). Moreover, RUNX2 protein levels were significantly reduced in all KH-treated groups (200 KH, 400 KH, and 600 KH) relative to the NC group (*p* < 0.05) (Figure 6C).

### 3.6. Osteogenic Bone Markers

**Figure 7:**
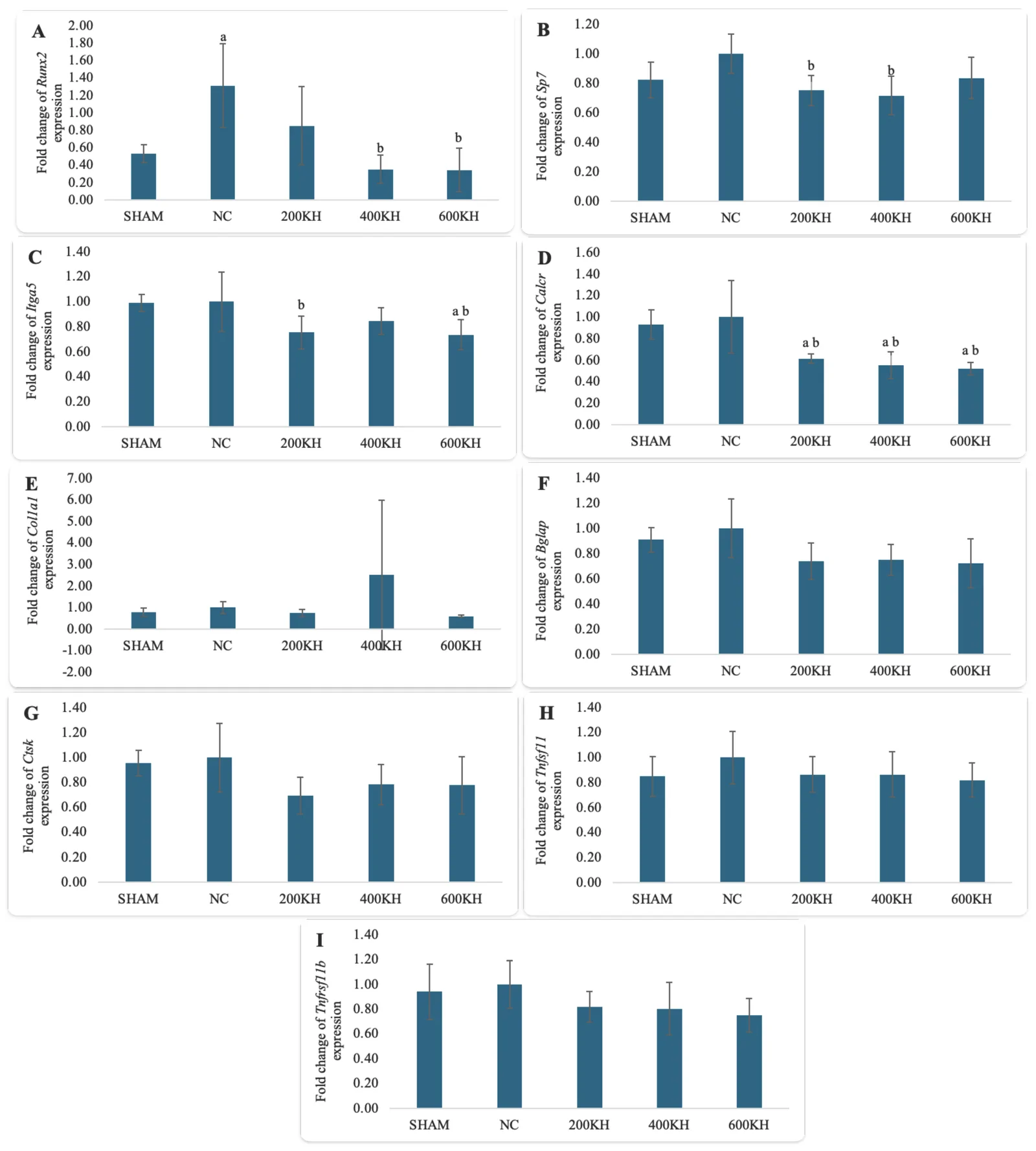
Osteogenic bone markers. Data are expressed as mean ± SD (*n* = 7 per group). Statistical significance was evaluated by one-way ANOVA followed by Tukey’s HSD multiple-comparison test. ^a^ *p* < 0.05 vs. SHAM, ^b^ *p* < 0.05 vs. NC.

Analysis of osteogenic and osteoclastogenic gene expression revealed several significant changes among the experimental groups. *Runx2* mRNA expression was significantly increased in the NC group compared with the sham group (*p* < 0.05), whereas the 400 KH and 600 KH groups exhibited significantly lower *Runx2* expression than the NC group (*p* < 0.05) (Figure 7A). Osterix (*Sp7*) mRNA expression was significantly reduced in the 200 KH and 400 KH groups relative to the NC group (*p* < 0.05) (Figure 7B). Similarly, Integrin Subunit Alpha 5 (*Itga5*) mRNA expression was significantly decreased in the 200 KH group compared with the NC group (*p* < 0.05), while the 600 KH group exhibited significantly lower *Itga5* expression than both the sham and NC groups (*p* < 0.05) (Figure 7C). Calcitonin receptor (*Calcr*) mRNA expression was significantly reduced in all KH-treated groups (200 KH, 400 KH, and 600 KH) compared with both the sham and NC groups (*p* < 0.05) (Figure 7D). In contrast, no significant differences were observed in the mRNA expression levels of *Col1a1*, bone gamma-carboxyglutamate protein (*Bglap*), cathepsin k (C*tsk*), receptor activator of nuclear factor κB ligand (*Tnfsf11*) and *Tnfrsf11b* among the experimental groups (*p* > 0.05) (Figure 7E–I).

## 4. Discussion

The negative control (NC) group exhibited increased bodyweight and impaired bone health, characterized by reduced bone mineral density, load, stress, and Young’s modulus, together with increased strain, displacement, CTX-1, RANK, and RUNX2 protein levels. KH supplementation improved body weight and several aspects of bone health, with the 200 KH group showing better weight outcomes and the 400 KH group demonstrating enhanced trabecular microarchitecture, as evidenced by increased Tb.N, Conn.D and Ct.Ar/Tt.Ar and reduced Tb.Th. Furthermore, KH supplementation improved bone biomechanical properties by increasing load, stress, and Young’s modulus while reducing displacement relative to the NC group. Kelulut honey also attenuated oxidative stress by increasing GPx and SOD activities in a dose-dependent manner, with CAT activity elevated in the 600 KH group. In addition, KH treatment modulated bone remodeling markers, reducing RUNX2 protein levels and *Calcr* gene expression, while altering the expression of CTX-1, RANK, *Runx2*, *Sp7*, and *Itga5*. Trabecular number, SMI, and the expression of *Col1a1*, *Bglap*, *Ctsk*, *Rankl*, and *Tnfrsf11b* were not significantly affected by KH supplementation.

The greater body weight gain observed in the NC group compared with the sham group confirms the obesogenic effect of prolonged HCHF feeding, which promotes excessive adiposity and metabolic dysfunction. The attenuation of body weight gain by KH supplementation may be attributed to the synergistic actions of several bioactive metabolites identified by LC-MS/MS analysis [9]. Notably, GABA has been reported to reduce obesity in six-weeks old male C57BL/6J mice by reducing WAT deposition and increasing iWAT beiging [11]. A systematic review and meta-analysis also provided further evidence that supplemental betaine could reduce body fat in human subjects [12]. Collectively, these metabolites may act synergistically to improve energy metabolism and reduce fat accumulation in HCHF-fed rats. Interestingly, the greatest reduction in body weight was observed in the 200 KH group rather than the higher-dose groups. This finding suggests a possible hormetic or biphasic dose-response, in which moderate doses achieve optimal metabolic efficacy, whereas increasing the dose does not proportionally enhance the biological response. Nevertheless, further mechanistic studies are required to determine which metabolites are primarily responsible for the anti-obesity effects of KH.

Micro-computed tomography (micro-CT) is a well-established technique for evaluating bone quality, enabling detailed three-dimensional assessment of cortical and trabecular bone microarchitecture at high resolution [13,14]. The microarchitectural parameters generated by micro-CT have been shown to correlate closely with those obtained through histomorphometric analysis [15]. In the present study, HCHF diet did not significantly affect bone microarchitecture, corroborating previous findings where there was no detectable microarchitectural changes following 16 weeks of HCHF diet administration [2,9]. Likewise, Wong et al. [16] found that a 20-week HCHF diet had no effect on Tb.N, Tb.Th, or Tb.Sp, despite a reduction in BV/TV. Contrary to our findings, Alonso et al. [17] observed significant impairments in BV/TV, Tb.Th, and Tb.N in rats subjected to a high-fat diet (HFD) for 10 months. This discrepancy may indicate that the detrimental effects of a high-fat diet on bone microarchitecture become more pronounced with longer durations of dietary exposure. Interestingly, KH supplementation improved several aspects of bone microarchitecture in HCHF-fed rats. The 400 mg/kg dose was associated with higher Tb.N, Conn.D and Ct.Ar/Tt.Ar, suggesting that the trabecular network became denser and more interconnected, characteristics associated with greater structural integrity and resistance to fracture. Likewise, the higher Ct.Ar/Tt.Ar ratio indicates preservation or enhancement of cortical bone mass, which contributes substantially to whole-bone mechanical strength [18]. Interestingly, although Tb.N was significantly higher in the 400 KH group than in the 200 KH group, Tb.Th was reduced. This finding may reflect a remodeling pattern in which bone formation preferentially generates a larger number of thinner trabeculae rather than fewer thicker trabeculae. Such an architectural adaptation is not necessarily detrimental because increased trabecular number and connectivity often compensate for reduced trabecular thickness, resulting in a more mechanically efficient trabecular network. The concurrent increase in Conn.D further supports the notion that the newly formed trabeculae remained well integrated within the trabecular lattice rather than existing as isolated structures. These findings suggest a dose-dependent effect of KH on trabecular architecture, with the higher dose promoting trabecular preservation primarily by increasing trabecular number and network connectivity rather than trabecular thickening. This remodeling pattern may enhance bone quality by maintaining the continuity of the trabecular framework, although the biological mechanisms underlying this response require further investigation.

Bone quality is determined not only by microarchitecture but also by its mechanical competence, which reflects the ability of bone to resist deformation and fracture [19]. Load and stress are the maximum forces a bone can withstand before failure and serve as indicators of bone strength. In contrast, stiffness and Young’s modulus describe the resistance of bone to elastic deformation, while displacement and strain reflect its capacity to deform prior to fracture, thereby providing measures of bone ductility [20]. Previous studies have reported varying effects of obesogenic diets on bone mechanical properties. Ekeuku et al. [2,9] observed reductions in displacement and strain, accompanied by increased stiffness [9], following 16 weeks of HCHF diet. In contrast, Alonso et al. [17] reported a decrease in load-bearing capacity in rats fed a high-fat diet for 10 months, while stress and Young’s modulus remained unchanged. These discrepancies may be attributed to differences in dietary composition and duration of exposure, suggesting that the skeletal response to HCHF/HFD consumption may evolve over time and become more pronounced following prolonged dietary intervention. In the present study, the HCHF diet adversely affected bone mechanical properties, as evidenced by reductions in load, stress, and Young’s modulus, accompanied by increased strain and displacement in the NC group. These changes may indicate a decline in bone strength and rigidity, resulting in bones that are less resistant to fracture and more susceptible to deformation under applied force. The biomechanical effects of KH observed in the present study differ somewhat from our previous findings. Ekeuku et al. [9] reported that supplementation with 1000 mg/kg KH for 8 weeks increased displacement and strain while reducing stiffness in HCHF-fed rats, suggesting an improvement in bone ductility. In contrast, the current study demonstrated increased stiffness, reduced displacement, and enhanced load- and stress-bearing capacity following treatment with lower doses of KH (200–600 mg/kg) for 12 weeks. These differences may be attributable primarily to variations in KH dosage, although differences in treatment duration may also have contributed to the observed outcomes. The higher dose previously used appeared to favor greater bone ductility, whereas the lower doses used in the present study were associated with improvements in bone strength and rigidity. Nevertheless, both studies consistently indicate that KH ameliorates HCHF-induced alterations in bone mechanical properties. Collectively, these findings suggest that the skeletal effects of KH may be dose-dependent, with different dosing regimens influencing distinct aspects of bone mechanical behavior, including strength, stiffness, and ductility. Further studies directly comparing different KH doses and treatment durations are warranted to elucidate the optimal regimen for improving bone mechanical properties under HCHF conditions.

BMD of the left femur was assessed using DXA, with BMD calculated as the ratio of bone mineral content to bone area [21]. Wong et al. [16] and Ekeuku et al. [9] reported no significant changes in BMD following 20 and 16 weeks of HCHF diet, respectively. Furthermore, it was found that supplementation with 1000 mg/kg KH for 8 weeks did not significantly affect BMD in HCHF-fed rats [9]. In contrast, the present study demonstrated that prolonged HCHF exposure resulted in significantly lower BMD in the NC group compared with the sham group at week 24, while supplementation with 600 mg/kg KH attenuated this decline. Longitudinal analysis revealed distinct patterns of BMD change among the experimental groups. The sham group exhibited a steady increase in BMD throughout the study period, reflecting normal skeletal growth and mineral accrual. Although BMD initially increased in the NC group, it subsequently declined, resulting in significantly lower values than those observed in the sham group by week 24. This pattern suggests that prolonged HCHF feeding may compromise bone mass maintenance. A similar trend was observed in the 200 KH group, whereas the 400 KH and 600 KH groups showed sustained increases in BMD over time. The discrepancy between the present findings and the one reported previously [9] may be due to differences in the duration and dosage of KH supplementation. While a higher dose administered for 8 weeks did not influence BMD, the current findings suggest that a longer treatment period may be required for the beneficial effects of KH on bone mineralization to become evident. Collectively, these results indicate that KH supplementation may help preserve BMD during prolonged HCHF exposure, with the greatest protective effect observed at the highest dose tested in the present study.

Prolonged consumption of HFD is known to increase the production of reactive oxygen species (ROS), leading to oxidative stress and disruption of the antioxidant defense system [22]. Excessive ROS has been implicated in obesity-related bone deterioration by suppressing osteoblast function and stimulating osteoclast activity, ultimately disrupting bone remodeling [23]. The primary defense against ROS is the first-line antioxidant defense system, which comprises enzymatic antioxidants including SOD, CAT and GPx. These enzymes act cooperatively to neutralize ROS and protect tissues from oxidative damage [24]. Consistent with impaired antioxidant defense, HCHF diet in the present study significantly reduced GPx and SOD activities after 24 weeks, indicating compromised antioxidant capacity. This observation contrasts previous finding which reported no significant changes in CAT or SOD following 16 weeks of HCHF feeding [9]. The contrasting findings may reflect differences in dietary exposure duration and the progressive nature of oxidative stress associated with prolonged metabolic disturbances. Interestingly, the effects of KH on antioxidant status also differed from those reported previously where KH supplementation improved skeletal outcomes without significantly affecting endogenous antioxidant markers [9]. Based on those findings, it was proposed that KH exerted its protective effects primarily by providing exogenous antioxidants derived from its bioactive constituents. In the present study, however, KH supplementation significantly increased GPx and SOD activities in a dose-dependent manner, with the greatest effects observed at the highest dose, whereas CAT activity was significantly elevated only at that dose. These findings indicate that KH enhances endogenous antioxidant defenses in addition to providing direct antioxidant effects. The longer duration of KH supplementation in the current study (12 weeks versus 8 weeks) may have provided sufficient time for modulation of antioxidant enzyme systems. Taken together, the findings suggest that the protective effects of KH against HCHF-induced bone deterioration may involve both direct antioxidant activity and the upregulation of endogenous antioxidant defenses, with the latter becoming more apparent following prolonged supplementation.

Bone tissue is continuously remodeled through the balanced actions of osteoclasts and osteoblasts, which regulate bone resorption and formation, respectively. Disruption of this balance can compromise skeletal integrity and contribute to the development of osteoporosis and other metabolic bone disorders [25]. In the present study, prolonged HCHF feeding increased both RUNX2 mRNA and protein levels, together with elevated RANk and CTX-1 levels. The increases in RANk and CTX-1 suggest enhanced osteoclastogenesis and bone resorption [26], whereas the upregulation of RUNX2 may represent a compensatory attempt to maintain or restore osteogenic activity in response to chronic HCHF-induced metabolic stress. As a key regulator of osteoblast differentiation and skeletal development [27,28], RUNX2 is often upregulated during periods of active bone remodeling. The observed changes are consistent with previous studies demonstrating that the skeletal response to obesogenic diets is dynamic and time dependent. Cai et al. [29] reported an initial reduction in RUNX2/*Runx2* expression after 4 weeks of HFD feeding, followed by an increase at 6 weeks, while Tian et al. [30] observed elevated *Runx2* and *Ctsk* expression after 8 and 16 weeks of HFD exposure, with both markers declining after 24 weeks. These findings suggest that prolonged HCHF/HFD exposure may be associated with changes in bone formation- and resorption-related markers, although direct effects on bone remodeling activity cannot be established from the present findings. Notably, although RANk and CTX-1 levels were elevated in the present study, *Ctsk* expression remained unchanged, indicating that the increased resorptive activity may have been driven primarily by enhanced osteoclast differentiation rather than changes in osteoclast enzymatic activity. Alternatively, *Ctsk* may be regulated differently during the later stages of metabolic bone deterioration. Despite increased RUNX2/*Runx2*, HCHF-fed rats exhibited reduced BMD, impaired biomechanical properties, and compromised antioxidant status, indicating that the osteogenic response was insufficient to compensate for the heightened resorptive activity. Furthermore, the absence of significant changes in other osteogenic markers, including *Col1a1*, *Bglap*, and *Sp7*, suggests that the increase in RUNX2 did not translate into a fully effective bone-forming response. Collectively, these findings indicate that prolonged HCHF feeding promotes a state of high bone turnover, in which compensatory osteoblast activation is unable to fully counteract excessive osteoclast-mediated bone resorption. KH supplementation was associated with changes in several markers related to bone formation and remodeling in HCHF-fed rats. HCHF-induced increases in *Runx2* mRNA and RUNX2 protein expression were reduced following KH supplementation, particularly at the higher doses. This reduction in *Runx2* following honey supplementation may reflect attenuation of the metabolic or cellular stress induced by HCHF feeding, thereby reducing the need for compensatory upregulation of osteogenic signaling. Similar reductions were observed in the expression of *Sp7*, *Itga5*, and *Calcr*, suggesting that KH may influence molecular pathways associated with osteogenic differentiation and bone remodeling. However, serum CTX-1 remained elevated in all KH-treated groups, while serum RANK levels were increased in the 200 KH and 400 KH groups but not in the 600 KH group. These findings indicate that the effects of KH on bone-related molecular and circulating turnover markers were not uniform. Nevertheless, KH was associated with improvements in bone mineral density, selected microarchitectural and biomechanical parameters, and antioxidant status. The differences observed between circulating bone turnover markers and tissue-level gene expression may reflect the complexity of skeletal responses to prolonged HCHF exposure. However, given the absence of direct histomorphometric assessment, the present findings cannot establish whether KH directly alters osteoblast or osteoclast activity or definitively normalizes bone remodeling. Notably, the absence of a further increase in RANk expression at the highest KH dose may indicate a differential response to KH treatment; however, this finding alone does not provide sufficient evidence for normalization of osteoclastogenic signaling. Collectively, the observed changes in oxidative status, bone quality, and bone turnover markers suggest that KH may influence multiple aspects of skeletal health, although the relative contribution of these pathways and the underlying mechanisms remain to be established.

An important observation in the present study was that the response to KH supplementation was not uniformly dose dependent across the measured endpoints. Although GPx and SOD activities increased progressively with KH dose, several skeletal parameters showed greater improvement at 400 mg/kg than at 600 mg/kg, suggesting a non-linear dose-response with an intermediate dose optimum. The lower response at 600 mg/kg should not necessarily be interpreted as an inhibitory effect, as this dose continued to confer beneficial effects on several skeletal outcomes. This non-monotonic response may be consistent with hormesis [31]; however, the present study was not specifically designed to establish a hormetic response. Further dose-ranging studies are warranted to determine whether the intermediate-dose optimum represents a reproducible hormetic pattern.

From a translational perspective, the KH doses used in the present study correspond to potentially achievable human-equivalent amounts. Using body-surface-area conversion, with a Km factor of 6 for rats and 37 for adult humans [32], the 200, 400, and 600 mg/kg rat doses correspond to approximately 32.4, 64.9, and 97.3 mg/kg in humans, respectively. For a 60-kg adult, these doses would correspond to approximately 1.95, 3.89, and 5.84 g of KH per day. Thus, the estimated human-equivalent amounts are within a practically achievable range for oral honey consumption. Nevertheless, these calculations should be interpreted cautiously, as body-surface-area conversion does not account for interspecies differences in absorption, metabolism, bioavailability, phytochemical composition, or pharmacokinetics. Furthermore, the present animal findings cannot be directly extrapolated to human efficacy or safety. Clinical studies are therefore required to determine whether comparable KH intake produces beneficial effects on bone health in humans and to establish an appropriate dose and duration of supplementation.

Several limitations of the present study should be acknowledged. First, bone remodeling was evaluated using selected serum biomarkers and gene expression analyses without histomorphometry assessment. Consequently, direct quantification of osteoblast and osteoclast number and activity within bone tissue was not possible. Second, molecular and biochemical parameters were assessed only at the end of the experimental period. Given the dynamic nature of bone remodeling, the temporal changes in antioxidant status and bone turnover markers throughout HCHF exposure and KH treatment could not be determined. Additionally, although KH supplementation improved bone mineral density, microarchitecture, biomechanical properties, and antioxidant status, serum CTX-1 and RANk levels remained elevated in some treatment groups. This apparent discrepancy suggests a complex relationship between bone turnover markers and skeletal outcomes and warrants further investigation to clarify the mechanisms underlying KH’s protective effects on bone health. Finally, although changes in antioxidant enzyme activities suggest alterations in antioxidant status, additional markers of oxidative damage, such as malondialdehyde and 8-hydroxy-2′-deoxyguanosine, as well as assessment of nuclear factor erythroid 2–related factor 2/heme oxygenase-1 (Nrf2/HO-1) signaling and NRF2 nuclear localization, were not included in the present study. Therefore, the involvement of oxidative stress and specific antioxidant signaling pathways in the effects of HCHF feeding and KH supplementation cannot be established definitively. Future studies incorporating direct measures of oxidative damage and molecular assessment of Nrf2/HO-1 signaling would provide further mechanistic insight into the bone-protective effects of KH under HCHF dietary conditions.

## 5. Conclusions

The present study demonstrates that prolonged HCHF diet consumption is associated with compromised skeletal health, characterized by reduced BMD and biomechanical strength, impaired antioxidant status, and alterations in bone remodeling-related markers. These changes were accompanied by decreased GPx and SOD activities, increased serum CTX-1 and RANk levels, and elevated *Runx2* expression. KH supplementation was associated with attenuation of several of these alterations, including improved trabecular microarchitecture, preservation of BMD, enhancement of biomechanical properties, and strengthening of antioxidant defenses. GPx and SOD activities increased progressively with KH dose, while CAT activity was significantly elevated at 600 mg/kg. KH supplementation was also associated with reduced expression of several bone remodeling-related genes, including *Runx2*, *Sp7*, *Itga5*, and *Calcr*. Although serum CTX-1 and RANk levels remained elevated in some treatment groups, the overall improvements in skeletal structure and mechanical properties coincided with improved antioxidant status and modulation of bone remodeling-related markers. The 400 and 600 mg/kg doses produced the most consistent skeletal and antioxidant benefits in the present model. Collectively, these findings support an association between KH supplementation, enhanced antioxidant status, modulation of bone remodeling-related markers, and preservation of skeletal health under HCHF-induced metabolic stress. However, direct mechanistic causality cannot be established from the present findings, and further studies incorporating histomorphometry, longitudinal assessment of bone turnover, direct measures of oxidative damage, and molecular pathway analyses are warranted to clarify the mechanisms underlying the observed skeletal effects of KH.

## Figures and Tables

**Table 1:** Target genes analysed and their corresponding NCBI Gene IDs.

Full Gene Name	Gene Symbol	Species	NCBI Gene ID
Runt-related transcription factor 2	*Runx2*	*Rattus norvegicus*	367218
Bone gamma-carboxyglutamate protein (Osteocalcin)	*Bglap*	*Rattus norvegicus*	25295
Tumor necrosis factor ligand superfamily member 11	*Tnfsf11 (Rankl)*	*Rattus norvegicus*	117516
Osteoprotegerin	*Tnfrsf11b (Opg)*	*Rattus norvegicus*	25341
Cathepsin K	*Ctsk*	*Rattus norvegicus*	29175
Collagen type I alpha 1 chain	*Col1a1*	*Rattus norvegicus*	29393
Integrin Subunit Alpha 5	*Itga5*	*Rattus norvegicus*	315346
Osterix	*Sp7*	*Rattus norvegicus*	300260
Calcitonin receptor	*Calcr*	*Rattus norvegicus*	116506
Glyceraldehyde-3-phosphate dehydrogenase	*Gapdh*	*Rattus norvegicus*	24383

Gene symbols and NCBI Gene IDs correspond to *Rattus norvegicus* and were obtained from the National Center for Biotechnology Information (NCBI) Gene database. GAPDH was used as the endogenous reference gene for normalization of mRNA expression.

## Data Availability

The original contributions presented in this study are included in the article and Supplementary Material. Further inquiries can be directed to the corresponding author.

## Supplementary Materials

The following supporting information can be downloaded at: https://www.mdpi.com/article/10.3390/life16091419/s1, Table S1: Previously reported phytochemical composition of the Kelulut honey sample used in the present study.
