# Supplementary material for: Kelulut Honey Modulates Skeletal Health and Bone-Related Molecular Markers in Rats Fed a High-Carbohydrate and High-Fat Diet
Source: Life (Basel). 2026 Aug 26;16(9):1419. doi: 10.3390/life16091419 (PMC13608747; doi:10.3390/life16091419)
Supplement: Supplementary file 1 [file life-16-01419-s001.zip › life-4493902-supplementary.pdf]

Table S1. Previously reported phytochemical composition of the Kelulut honey sample used in the present study.

No	Retention time (min)	m/z	Adduct	Molecular weight (Da)	PubChem Name	CID	Formula
1	0.485	104.07100	[M+H]+	103.06316	Gamma-aminobutyric acid	119	C4H9NO2
2	0.522	104.10725	[M]+	104.10725	Choline	305	C5H14NO
3	0.472 (0.476)	106.05018 (104.03429)	[M+H]+ ([M-H]-)	105.04234 (105.04213)	Serine	5951	C3H7NO3
4	2.976	118.06545	[M+H]+	117.05761	Indole	798	C8H7N
5	0.69	118.08644	[M+H]+	117.07860	Valine	1182	C5H11NO2
6	0.553	118.08648	[M+H]+	117.07864	Betaine	247	C5H11NO2
7	0.697	136.06166	[M+NH4]+	118.02320	Methylmalonic acid	487	C4H6O4
8	0.499 (0.496)	120.06594 (118.04964)	[M+H]+ ([M-H]-)	119.05810 (119.05748)	Threonine	205	C4H9NO3
9	3.064	122.08775	[M+H]+	121.07991	Phenethylamine	1001	C8H11N
10	1.069	123.04432	[M+H]+	122.03648	Benzoic acid	243	C7H6O2
11	0.651	125.02330	[M-H]-	126.03114	1,2,4-Benzenetriol	10787	C6H6O3
12	21.815	127.03920	[M+H]+	126.03136	4-Hydroxy-6-methyl-2-pyrone	54675757	C6H6O3
13	0.499	130.05013	[M+H]+	129.04229	Pyroglutamic acid	7405	C5H7NO3
14	0.735	130.08636	[M+H]+	129.07852	Pipecolic acid	849	C6H11NO2
15	1.301	132.10193	[M+H]+	131.09409	Isoleucine	6306	C6H13NO2
16	0.489	132.02922	[M-H]-	133.03706	Aspartic acid	5960	C4H7NO4
17	0.581	138.05490	[M+H]+	137.04706	Trigonelline	5570	C7H7NO2
18	2.976	120.08118	[M-H2O+H]+	137.08862	Phenylethanolamine	1000	C8H11NO
19	21.94	143.03418	[M+H]+	142.02634	Kojic acid	3840	C6H6O4
20	3.595	146.06030	[M+H]+	145.05246	Indole-3-Carboxaldehyde	10256	C9H7NO
21	0.499 (0.489)	147.07651 (145.06079)	[M+H]+ ([M-H]-)	146.06867 (146.06863)	Glutamine	5961	C5H10N2O3
22	0.425	147.11292	[M+H]+	146.10508	Lysine	5962	C6H14N2O2
23	0.913	146.11766	[M]+	146.11766	Acetylcholine	187	C7H16NO2
24	0.693	146.04465	[M-H]-	147.05249	N-Acetyl-DL-serine+G31	352294	C5H9NO4

Data summarized from [Ekeuku et al. 2025]. The phytochemical composition of the KH sample was previously characterized in this publication.
